# Maltodextrin enhances biofilm elimination by electrochemical scaffold

**DOI:** 10.1038/srep36003

**Published:** 2016-10-26

**Authors:** Sujala T. Sultana, Douglas R. Call, Haluk Beyenal

**Affiliations:** 1 School of Chemical Engineering & Bioengineering, Washington State University, Pullman, 99164, WA, USA; 2 Paul G. Allen School for Global Animal Health, Washington State University, Pullman, 99164, WA, USA

## Abstract

Electrochemical scaffolds (e-scaffolds) continuously generate low concentrations of H_2_O_2_ suitable for damaging wound biofilms without damaging host tissue. Nevertheless, retarded diffusion combined with H_2_O_2_ degradation can limit the efficacy of this potentially important clinical tool. H_2_O_2_ diffusion into biofilms and bacterial cells can be increased by damaging the biofilm structure or by activating membrane transportation channels by exposure to hyperosmotic agents. We hypothesized that e-scaffolds would be more effective against *Acinetobacter baumannii* and *Staphylococcus aureus* biofilms in the presence of a hyperosmotic agent. E-scaffolds polarized at −600 mV_Ag/AgCl_ were overlaid onto preformed biofilms in media containing various maltodextrin concentrations. E-scaffold alone decreased *A. baumannii* and *S. aureus* biofilm cell densities by (3.92 ± 0.15) log and (2.31 ± 0.12) log, respectively. Compared to untreated biofilms, the efficacy of the e-scaffold increased to a maximum (8.27 ± 0.05) log reduction in *A. baumannii* and (4.71 ± 0.12) log reduction in *S. aureus* biofilm cell densities upon 10 mM and 30 mM maltodextrin addition, respectively. Overall ~55% decrease in relative biofilm surface coverage was achieved for both species. We conclude that combined treatment with electrochemically generated H_2_O_2_ from an e-scaffold and maltodextrin is more effective in decreasing viable biofilm cell density.

*Acinetobacter baumannii* and *Staphylococcus aureus* are important nosocomial pathogens that are commonly found in biofilm-infected wounds of long-term, acute-care patients^1^,^2^,^3^. Antibiotic treatment often does not work against biofilm communities because of their protective biofilm matrix^4^; consequently, alternative antimicrobial “scaffolds” have been developed that incorporate silver, iodide, zinc, honey, or other polysaccharide substance like glycol to treat biofilm infections^5^,^6^,^7^,^8^,^9^. Nevertheless, no existing scaffolds are capable of the continuous, controlled delivery of antimicrobials for the complete eradication of biofilm infections. A recently developed electrochemical scaffold (e-scaffold) produces a continuous, localized, low concentration of H_2_O_2_ near the biofilm surface that is sufficient to damage biofilm communities with no apparent damage to host tissue^10^. The e-scaffold functions by partially reducing dissolved oxygen in aqueous solution to form H_2_O_2_ as per equation (1)^10^,^11^.


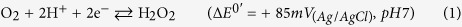


This reaction requires a negative polarization potential^12^. Based on this finding, an e-scaffold was developed using a conductive carbon fabric material that can be overlaid onto biofilm-infected surfaces^10^. When polarized at −600 mV_Ag/AgCl_, the e-scaffold reduces oxygen to produce a sustained concentration of H_2_O_2_ near the fabric surface, which can prevent/delay biofilm growth or remove preformed biofilms^10^,^13^. In practical terms, an e-scaffold saturated with an electrolyte can be overlaid on the biofilm-infected wound surface to keep it moist and electrochemically reduce the dissolved oxygen to H_2_O_2_^10^. Although this previously developed e-scaffold prevented/delayed or removed biofilm growth, its efficacy can be improved and this is the goal of the present work.

H_2_O_2_ damages bacterial DNA and kill bacterial cells by causing irreversible oxidative damage to the thiol groups of bacterial proteins and lipids^14^,^15^,^16^,^17^,^18^. Nevertheless, the efficacy of H_2_O_2_ is dependent on how the bacterial population responds to oxidative stress and this can differ for Gram-negative and Gram-positive bacteria^19^,^20^,^21^,^22^. The entry of H_2_O_2_ into bacterial cells can be limited as a function of lipid composition, diffusion-facilitating channel proteins, or both^23^,^24^. Furthermore, the presence of catalase can decompose H_2_O_2,_ and thus catalase effectively serves as a permeability barrier for the bacterial cell^25^,^26^,^27^. The decomposition of H_2_O_2_ by catalase in the biofilm matrix was considered a limiting mechanism for e-scaffolds. Nevertheless, recent work has shown that when the H_2_O_2_ is delivered continuously at low concentrations (on the order of μM), H_2_O_2_ can diffuse into biofilms faster than it decomposes and thus it can be used as an effective biocide at low concentrations^10^,^13^.

The rate of H_2_O_2_ diffusion into a biofilm is controlled by characteristics of the biofilm such as its density and reactivity with H_2_O_2_^23^,^25^,^28^,^29^,^30^. Furthermore, when exposed to a negative potential (~−700 mV_Ag/AgCl_) bacterial cells respond by generating osmolytes, including trehalose, betaine, proline and glutamate, that can protect cells from external injuries^31^. These osmolytes likely scavenge e-scaffold-generated H_2_O_2_, retard its entry into bacteria and consequently decrease the efficiency of the system^32^,^33^,^34^,^35^.

It is possible to facilitate H_2_O_2_ entry into bacterial cells by activating bacterial membrane transportation channels^24^,^36^,^37^ in a low-osmolarity medium containing a hyperosmotic agent^38^,^39^,^40^. For example, bacteria can respond to conditions of low osmolarity by increasing the density of membrane porins, especially aquaporin^41^,^42^,^43^,^44^, which in turn can enhance H_2_O_2_ entry into cells^24^,^37^. A hyperosmotic agent at low osmolarity induces oxidative damage by altering gene expression, including increasing catalase expression may form non-membrane channels permitting water and H_2_O_2_^37^,^45^,^46^,^47^,^48^. It can also enhance H_2_O_2_ entry into cells by “stretching” the lipid bilayer^24^. Further increasing the osmolarity of the medium with a hyperosmotic agent, however, can eventually cause blockage of the transportation pathway^49^. In addition, at higher osmolarities bacteria synthesize more osmolytes that protect the cells by impeding antimicrobial entry^50^. Therefore, it is expected that there is an optimal hyperosmotic agent concentration for obtaining effective H_2_O_2_ entry.

An earlier investigation showed that an e-scaffold produces a constant supply of H_2_O_2_ (~25 μM) and that this concentration is sufficient to reduce *A. baumannii* populations by (4 ± 0.28) log for both *in vitro* biofilms and biofilm-infected porcine explants^10^. Based on our previous work with hyperosmotic agent treatments^49^ we hypothesized that operation of an e-scaffold in the presence of maltodextrin, a hyperosmotic agent, would be more effective against *A. baumannii* and *S. aureus* biofilms than treatment with either individual application alone. Maltodextrin is a product of hydrolyzed starch and is composed of sugars and polysaccharides. Besides its hypothesized benefits as a hyperosmotic agent, maltodextrin reportedly controls odor from infected wounds and ulcers while promoting the growth of highly vascularized granulation tissue in clinical trials^51^,^52^. Because osmotic responses can differ between Gram-positive and Gram-negative bacteria^40^,^46^, we expected the optimal concentration of maltodextrin would vary between *A. baumannii* and *S. aureus* biofilms. We treated biofilm samples with maltodextrin or e-scaffold alone or with combination of e-scaffold and maltodextrin and then quantified the changes in cell viability and biofilm surface coverage.

## Results

### Effect of maltodextrin and e-scaffold on cell recovery

**Figure 1:**
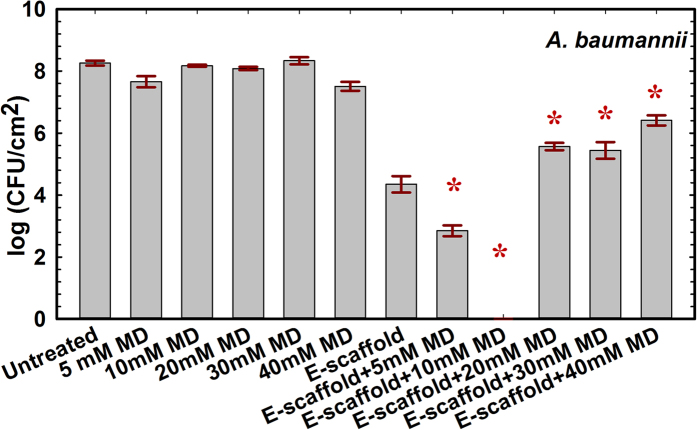
Maltodextrin enhances the efficacy of e-scaffold to eliminate viable *A. baumannii* biofilm cell density. Bars represent means of log (CFU/cm^2^) of viable biofilm cells for three biological replicates. Error bars represent the standard error of the means calculated from triplicate measurements. The symbol *denotes a significant difference compared to treatment with an e-scaffold alone (n = 3 and *P* < 0.001, one-way ANOVA with Bonferroni post hoc t-test). E-scaffolds were polarized at −600 mV_Ag/AgCl_ and the average current density was −56 μA/cm^2^.

Treatment with an e-scaffold alone reduced the viable *A. baumannii* biofilm cell density by (3.92 ± 0.15) log compared to that of untreated biofilms (Fig. 1). The addition of maltodextrin (5, 10, 20, 30 and 40 mM) changed the average CFU recovery from *A. baumannii* biofilms compared to that for e-scaffold treatment alone (one-way ANOVA, *P* < 0.001). This resulted in a “U-shaped” dose response with respect to log-counts of recovered bacteria (2.85 ± 0.17, 0, 5.57 ± 0.12, 5.44 ± 0.27 and 6.41 ± 0.16, respectively). We recovered no viable *A. baumannii* from biofilms treated with an e-scaffold and 10 mM maltodextrin. A (4.35 ± 0.16) log reduction of viable biofilm cell density compared to that for treatment with an e-scaffold alone indicates that the e-scaffold is more effective against *A. baumannii* biofilms when it is used in combination with 10 mM maltodextrin (one-way ANOVA, *P* < 0.001).

**Figure 2:**
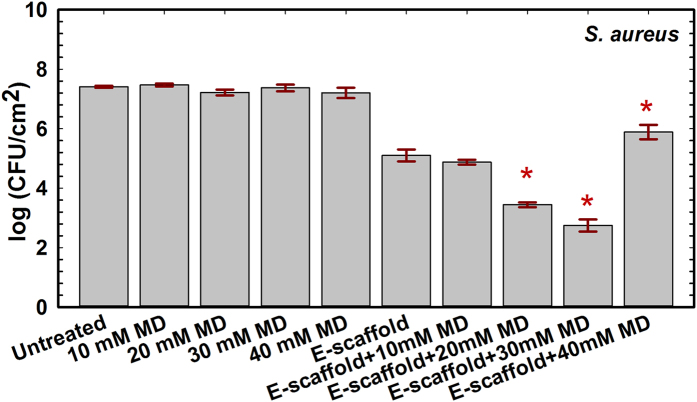
Maltodextrin enhances the efficacy of e-scaffold to eliminate viable *S. aureus* biofilm cell density. Bars represent means for three biological replicates. Error bars represent the standard errors of the means calculated from triplicate measurements. The symbol *denotes a significant difference compared to treatment with an e-scaffold alone (n = 3 and *P* < 0.001, one-way ANOVA with Bonferroni post hoc t-test). E-scaffolds were polarized at −600 mV_Ag/AgCl_ and the average current density was −60 μA/cm^2^.

The cell counts for e-scaffold-treated *S. aureus* biofilms decreased by (2.31 ± 0.12) log compared to those for untreated biofilms (Fig. 2). Compared to biofilms treated with an e-scaffold alone, the addition of 10 or 20 mM maltodextrin in combination with the e-scaffold resulted in a further decrease in log-count of 0.23 ± 0.12 or 1.66 ± 0.13, respectively (Fig. 2). The addition of 30 mM maltodextrin resulted in an additional (2.40 ± 0.17) log reduction in recoverable *S. aureus* compared to treatment with an e-scaffold alone (one-way ANOVA, *P* < 0.001).

Overall, the efficacy of the e-scaffold at reducing viable biofilm cell density was enhanced in low-osmolarity maltodextrin media (10 mM for *A. baumannii* and 30 mM for *S. aureus*). Among the treatment conditions, the combination of an e-scaffold and 10 mM maltodextrin achieved the maximum reduction in viable *A. baumannii* biofilm cell density, (8.27 ± 0.05) log (n = 3, one-way ANOVA, *P* < *0.*001) compared to untreated biofilms. The combination of an e-scaffold and 30 mM maltodextrin was found to be the most effective treatment against *S. aureus* biofilms with a (4.71 ± 0.12) log reduction in viable cell density (n = 3, one-way ANOVA, *P* < *0.*001) compared to untreated biofilms. For both strains, maltodextrin alone had no significant effect on viable biofilm cell density. Thus, the combination of an e-scaffold and maltodextrin was more effective against both biofilms than either individual treatment alone. *A. baumannii* showed the maximum sensitivity to H_2_O_2_ generated from an e-scaffold in combination with maltodextrin.

### Effects of maltodextrin and e-scaffold treatment on biofilm surface coverage

**Figure 3:**
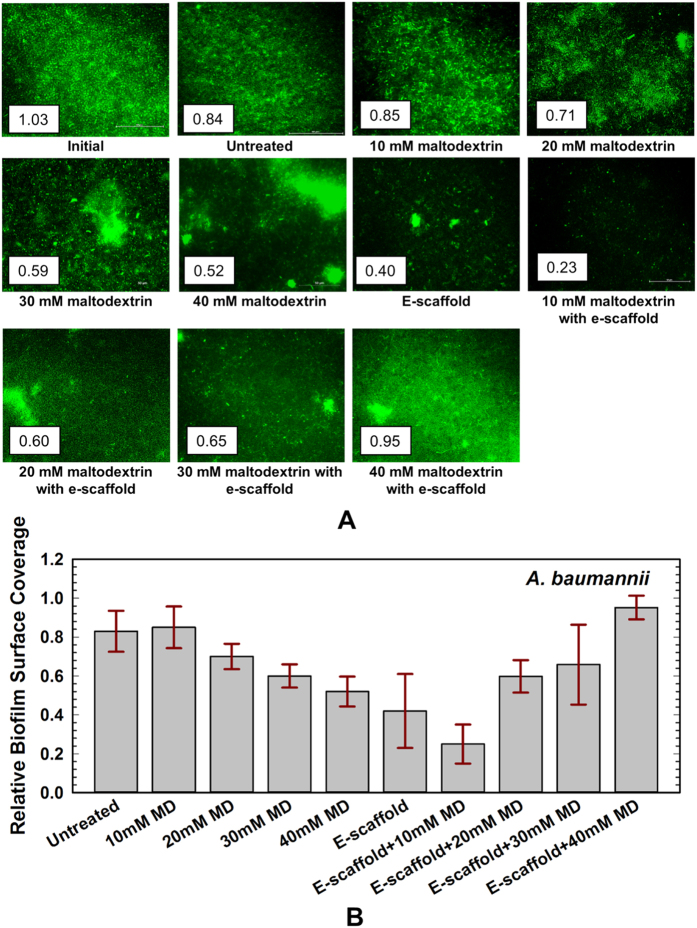
(**A**) *A. baumannii* biofilm grown for 1 day (initial biofilm) on glass bottom petridish, and all other images taken 24 h later for different treatment condition. One example image is presented for each condition along with their corresponding relative surface coverage; scale bar = 50 μm and 40x magnification. (**B**) Relative surface coverage for untreated biofilms and biofilms under varying treatment conditions of maltodextrin (MD) and e-scaffold. The concentrations tested were 36 mg/mL MD (10 mM), 72 mg/mL MD (20 mM), 108 mg/mL MD (30 mM) and 144 mg/mL MD (40 mM). Each biofilm was imaged ten times and the results were averaged (n = 3 independent replicates). Error bars represent the standard error of the mean. No statistically significant difference in biofilm surface coverage was observed between an e-scaffold with maltodextrin and an e-scaffold alone.

**Figure 4:**
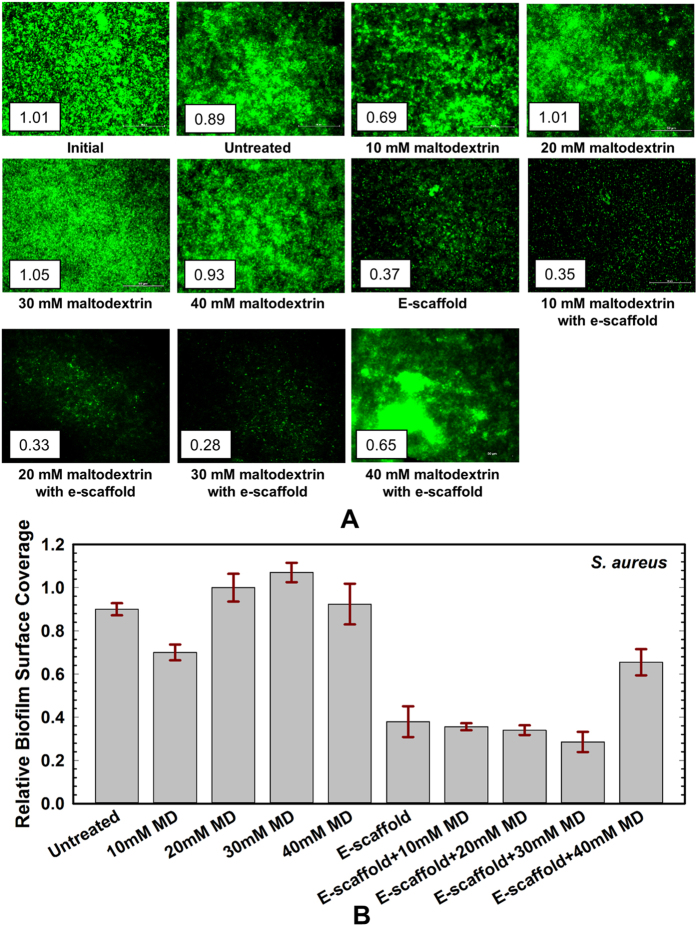
(**A**) *S. aureus* biofilm growth for 1 day (initial biofilm) on glass bottom petridish and 24 h thereafter for different treatment condition. One example image is presented for each condition with their corresponding relative surface coverage; scale bar = 50 μm and 40x magnification. (**B**) Relative surface coverage for untreated biofilms and biofilms under varying treatment conditions of maltodextrin (MD) and e-scaffold. The concentrations tested were 36 mg/mL MD (10 mM), 72 mg/mL MD (20 mM), 108 mg/mL MD (30 mM) and 144 mg/mL MD (40 mM). Each biofilm was imaged ten times and the results were averaged (n = 3 independent replicates). Error bars represent the standard error of the mean. No statistically significant difference in biofilm surface coverage was observed between an e-scaffold with maltodextrin and an e-scaffold alone.

After 24 h the untreated biofilms and maltodextrin and/or e-scaffold treated biofilms were compared by calculating average relative biofilm surface coverages from inverted microscope images (Figs 3 and 4). The addition of maltodextrin alone (10, 20, 30, or 40 mM) resulted in a dose-dependent reduction in the biofilm surface coverage, but it was not statistically significant compared to untreated biofilms. When *A. baumannii* biofilms were challenged with both an e-scaffold and 10 mM maltodextrin, maximum decrease in biofilm surface coverage (58.3 ± 8.4% compared to untreated biofilms) was observed (Fig. 3). This corresponds to an additional ~17% decrease compared to biofilms treated with an e-scaffold alone, but the difference is not statistically significant (*P* > 0.05, Student’s t-test). When we challenged the biofilm with a combination of e-scaffold and ≥ 20 mM maltodextrin, biofilm surface coverage increased compared to biofilms treated with an e-scaffold alone (Fig. 3).

When *S. aureus* biofilms were treated with maltodextrin alone there was no statistically significant effect on biofilms surface coverage compared to untreated biofilms (Fig. 4). When we challenged the biofilm with a combination of e-scaffold and maltodextrin (10, 20 and 30 mM), there was a statistically insignificant decrease in biofilm surface coverage compared to biofilms treated with an e-scaffold alone (Fig. 4B). Compared to biofilms treated with an e-scaffold alone, the addition of 40 mM maltodextrin resulted in an increase in biofilm surface coverage. Overall, the maximum decrease in *S. aureus* biofilm surface coverage (57.5 ± 3.2% compared to untreated biofilms) was observed with the combination of an e-scaffold and 30 mM maltodextrin. This corresponds to an additional ~9.5% decrease compared to the biofilms treated with an e-scaffold alone.

## Discussion

The diffusion of e-scaffold-generated H_2_O_2_ through the biofilm matrix and its entry through the bacterial membrane are dependent on the osmolarity of the medium^53^. With increasing maltodextrin concentration, the osmolarity of the solution increases^54^. At relatively high osmolarities, however, adaptive responses to osmotic stress will limit the rate of H_2_O_2_ entry into a cell^55^. Thus, a “U-shaped,” or “biphasic,” response should be expected when maltodextrin is used as the osmotic agent. We found that, in combination with an e-scaffold, 10 mM maltodextrin was the most effective against *A. baumannii*, while 30 mM maltodextrin was the most effective against *S. aureus* biofilms. Increasing the concentration of maltodextrin up to 40 mM in combination with an e-scaffold did not increase biofilm elimination. This may be due to blockage of H_2_O_2_ diffusion pathways through the biofilm matrix at higher concentrations of maltodextrin, since the relative diffusivity of an antimicrobial can decrease with increasing concentrations of maltodextrin, as observed previously^49^,^53^.

The image analysis shows that for both biofilms the maximum decrease in biofilm surface coverage (~55% decrease from that of the untreated biofilm) occurred when the biofilm was treated with the combination of an e-scaffold and maltodextrin. The effect on biofilm surface coverage, however, was not statistically significant compared to that for e-scaffold alone. This contrasts with the biofilm cell viability data, which showed a clear treatment benefit when maltodextrin was combined with an e-scaffold. Hence, changes in the biofilm surface coverage (as measured here) are unlikely to be the mechanism enhancing the efficacy of the e-scaffold. The most likely mechanism may be increased permeation of e-scaffold-generated H_2_O_2_ into the bacterial membrane due to changes induced by the addition of a low-osmolarity hyperosmotic agent^37^,^43^. For example, the overexpression of aquaporin proteins in Gram-negative bacteria has been reported in a low-osmolarity medium containing a hyperosmotic agent^33^,^35^,^36^,^37^ that facilitates H_2_O_2_ entry through membranes^38^,^50^. Gram-positive *S. aureus* has a thicker peptidoglycan layer^51^ and does not produce porins^32^,^38^, which is speculated to be the reason for its lesser sensitivity to this treatment compared to Gram-negative *A. baumannii*.

Antimicrobial “scaffolds” incorporating silver, honey, iodine or other substances have been reported as a promising alternative, antibiotic-free technology for multidrug-resistant bacteria, in particular for biofilm elimination from infected wound surfaces^7^,^56^. Nevertheless, inconsistent results have been obtained because of the uncontrolled delivery of the active antimicrobial agent^56^. For instance, there is a plethora of silver-impregnated dressings available commercially; these show a range of log-reductions of 0–6 for Gram-positive *S. aureus*, 0.2–8.4 for Gram-negative *Acinetobacter* spp. and 0.1–6.4 for *P. aeruginosa* PAO1 planktonic cultures^57^,^58^. The efficacy of these dressings against biofilms decreases to a range of 0–4 log-reduction for Gram-positive *S. aureus* and 0–2 log-reduction for Gram-negative *P. aeruginosa*^59^,^60^. The observed variation in log-reduction has been attributed to inconsistency in the concentration of silver ions released to biofilm^58^. Silver ions deactivate rapidly when released into the medium^58^. In addition, silver dressings have been reported to select for silver resistance in bacteria^61^ and they can cause cytotoxicity to fibroblast cells^56^,^58^. Medical-grade honey of 10–40% (v/v) or a honey-impregnated dressing is another alternative, reported to achieve around a 1–5 log-reduction for many Gram-positive and Gram-negative bacteria and biofilms^62^,^63^. The exact mechanisms of action of honey-impregnated dressings are still elusive^56^. Dressings impregnated with iodophores such as cadaxomer iodine and betadine have also been reported to be very effective in biofilm reduction to a range of 0–8 log^59^,^64^. Similar to silver, iodine reportedly has toxic effects and a limited timescale for its use^65^. Thus, none of the currently available antimicrobial dressings can deliver a constant, controlled concentration of antimicrobials to achieve a consistent efficacy in biofilm elimination.

Here, we proposed the combination of an e-scaffold and maltodextrin as an alternative biofilm elimination technology. This achieved an overall log-reduction of 8.27 ± 0.05 for Gram-negative *A. baumannii* and 4.71 ± 0.12 for Gram-positive *S. aureus* biofilms over 24 h, which is significantly more effective than reports for other antimicrobial technologies. Both H_2_O_2_ and maltodextrin are individually used in wound care^51^,^66^,^67^,^68^,^69^. We previously demonstrated that an e-scaffold generates a low concentration of H_2_O_2_ that can be an effective, nontoxic alternate treatment for biofilm-infected wounds^10^. Maltodextrin benefits wound healing by promoting collagen formation, granulation tissue growth and epithelial proliferation^52^,^69^,^70^,^71^. The combination of an e-scaffold and maltodextrin clearly enhances the elimination of biofilms of two nosocomial infectious agents, Gram-negative *A. baumannii* and Gram-positive *S. aureus*. In practice, the e-scaffold can be overlaid onto a biofilm-infected wound in conjunction with a maltodextrin gel or solution. Such treatment can eliminate biofilms while helping to maintain an environment favorable for wound healing^52^,^70^. Overall, our proposed technology offers enhanced effectiveness in biofilm-infected wound treatment.

## Materials and Methods

### Culture growth

Strains of *Acinetobacter baumannii* (ATCC #17978) and *Staphylococcus aureus* (ALC1743) expressing green fluorescent protein (gfp) were used in this study for fluorescence imaging purposes. *A. baumannii* was provided by Professor Eric P. Skaar of the Department of Pathology, Microbiology and Immunology, Vanderbilt University, Nashville, TN, and an *S. aureus* strain was provided by Niles Donegan of the Giesel School of Medicine at Dartmouth College, Hanover, NH. Cultures were grown as per published protocols^72^,^73^,^74^. Briefly, cultures were grown in 20 g/L (1×) Luria Broth (LB) medium (Sigma-Aldrich, catalog # L3522) supplemented with ampicillin (100 μg/mL; Sigma-Aldrich, catalog #A5354) and in 40 g/L (1×) tryptic soy broth (TSB) medium (Fisher Scientific, catalog #211825) supplemented with chloramphenicol (10 μg/mL, catalog #C1919-25G). All cultures were grown overnight at 37 °C at an agitation speed of 70 rpm on a rotary shaker.

### Biofilm growth

LB medium (0.05×) with ampicillin (100 μg/mL) was used for *A. baumannii* biofilm culture. TSB medium (0.1×) with chloramphenicol (10 μg/mL) was used for *S. aureus* biofilm culture. Overnight cultures were adjusted to OD_600_ ≈ 0.5 before use as inocula. Sterile glass-bottom petri dishes (MatTek Corporation, catalog #P35G-1.5-20-C) were inoculated with 2 ml of overnight cultures. After 2 h of initial attachment, suspended bacteria were removed by washing twice with fresh medium. Biofilms were allowed to develop on the glass surfaces for 24 h.

### Biofilm treatment with maltodextrin and e-scaffold

**Figure 5:**
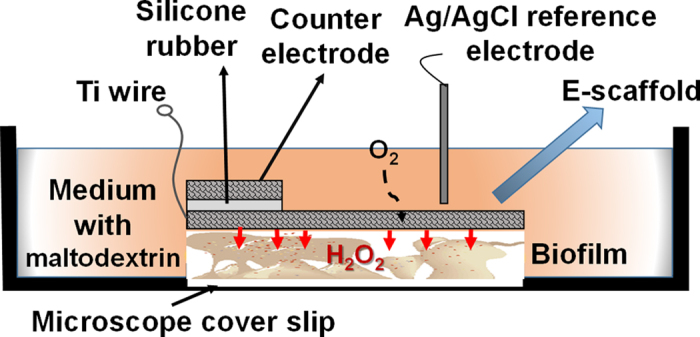
Schematic of experimental setup for the treatment of biofilm exposed to an e-scaffold with an illustration of electrochemical H_2_O_2_ production. The electrodes are connected to a potentiostat (not shown in figure). Microscope glass coverslips were used as biofilm growth surfaces.

The electrochemical scaffolds (e-scaffolds) consisted of three electrodes prepared as described in Supplementary information and shown in Fig. 5. Maltodextrin (Sigma Aldrich, catalog #419672) solutions were prepared in the respective growth media. After 24 h of growth, *A. baumannii* and *S. aureus* biofilms were imaged to collect baseline data. Fresh media (4 ml) with different final concentrations of maltodextrin (0, 5, 10, 20, 30, or 40 mM) were added back to the biofilms. The e-scaffolds were then placed on top of the preformed biofilms on the glass surfaces of the petri dishes (Fig. 5).

### Biofilm cell viability measurement

All biofilms exposed to an e-scaffold and/or maltodextrin were collected after 24 h and viable cells were enumerated. E-scaffolds were carefully removed and sonicated in 2 ml fresh medium for 30 s at 40 kHz with a power output of 72 W (Bransonic 1510R-MTH; Bransonic Ultrasonic Corp., Danbury, CT). Biofilms on the glass-bottom petri dishes were carefully washed twice with fresh medium to remove loosely attached cells. Biofilms/attached cells were then scraped off the glass surfaces and resuspended in 3 ml of fresh medium^10^. Cell suspensions (total 5 ml) recovered from e-scaffolds and glass-bottom petri dishes were mixed by vortexing for 30 s and were centrifuged (4,180× g for 10 min). Each resulting cell pellet was resuspended in 1 ml of medium, and serial dilutions were prepared. Colony forming units (CFU) of viable biofilm cells were quantified using a modified drop-plate method^75^. Untreated biofilms with non-polarized e-scaffolds were considered to be negative controls.

### Imaging and quantifying the biofilm structure

Cells expressing gfp were imaged using an inverted epifluorescence microscope (Nikon Eclipse Ti-S inverted microscope) with a Nikon DS-Qi1Mc camera mounted on it and a CFI Plan Fluor ELWD 40x objective (N.A. 0.60, W.D. 3.72.7 mm). Each biofilm was imaged after 24 h of growth before any treatment (initial biofilm, t = 0 h) and after 24 h of treatment. Biofilms were washed twice to remove any planktonic cells and refreshed with medium prior to imaging. The images were evaluated using Image Structure Analyzer (ISA) software^76^,^77^. At least ten discrete images were taken for each time point^13^,^78^. Biofilm surface coverage was defined as the ratio of the area of biofilm to the total area of the image. Biofilm surface coverage was normalized with the average surface coverage of initial biofilms and reported as relative biofilm surface coverage.

### Statistical analysis

At least three independent replicates were completed for each set of experimental conditions. Technical replicates were averaged before analysis using Student t-tests or one-way ANOVA with a Bonferroni pairwise test to identify differences between treatment groups (SigmaPlot, version 13, Systat Software, Inc., San Jose, CA).

## Conclusions

Our findings show that a combined treatment with electrochemically generated H_2_O_2_ and maltodextrin is more effective in decreasing viable biofilm cell density than either treatment alone. This combination also achieved the maximum decrease in biofilm surface coverage (~55% from the untreated biofilm coverage). Gram-negative *A. baumannii* biofilm cells were eradicated by the e-scaffold at a lower osmolarity (10 mM) maltodextrin solution, showing they are more sensitive to this treatment than Gram-positive *S. aureus* biofilms. In the case of *S. aureus* biofilms, the reduction in viable biofilm cell density by e-scaffold increased with increasing osmolarity and reached a maximum reduction at 30 mM maltodextrin. For both biofilms, after the maximum decrease in viable biofilm cell density was reached, further increase in the maltodextrin concentration reduced the treatment effectiveness, probably by blocking H_2_O_2_ diffusion. These results indicate the dose-dependent effect of maltodextrin in enhancing biofilm removal efficacy of e-scaffold. Overall, we found that the combined effect of maltodextrin and H_2_O_2_ produced by an e-scaffold enhanced biofilm elimination compared to treatment with either application alone. This combination completely eradicated one-day-old Gram-negative *A. baumannii* biofilms and produced a ~5 log-reduction in Gram-positive *S. aureus* biofilms, which is very effective biofilm elimination. Further clinical study would provide better evidence for the potential of this technology as a biofilm-infected wound treatment.

## Additional Information

**How to cite this article**: Sultana, S. T. *et al*. Maltodextrin enhances biofilm elimination by electrochemical scaffold. *Sci. Rep.* **6**, 36003; doi: 10.1038/srep36003 (2016).

**Publisher’s note:** Springer Nature remains neutral with regard to jurisdictional claims in published maps and institutional affiliations.

## Supplementary Material

Supplementary Information
