# Supplementary material for: Maltodextrin enhances biofilm elimination by electrochemical scaffold
Source: Sci Rep. 2016 Oct 26;6:36003. doi: 10.1038/srep36003 (PMC5080540; doi:10.1038/srep36003)
Supplement: Supplementary Information [file srep36003-s1.pdf]

Supplementary Information for

Maltodextrin enhances biofilm elimination by electrochemical scaffold

Sujala T Sultana[1], Douglas R Call[2], and Haluk Beyenal[1,*]

[1]The Gene and Linda Voiland School of Chemical Engineering and Bioengineering, [2]Paul G. Allen School for Global Animal Health, Washington State University, Pullman, Washington

Running title: Maltodextrin and e-scaffold eliminates biofilms

[*]Corresponding author:

Email: beyenal@wsu.edu; Telephone: +1-509-335-6607; Fax: +1-509-335-4806

Electrochemical scaffold preparation. The purpose of the working electrode of the electrochemical scaffold (e-scaffold) was to hold a negative polarity to reduce atmospheric oxygen and generate H_2O_2 (Sultana et al. 2015). To complete the electrochemical cell, we used a counter electrode and a custom-made Ag/AgCl reference electrode. Briefly, a custom-built e-scaffold was fabricated using carbon fabric (Panex 30 PW-06, Zoltex Companies Inc., St Louis, MO). The fabric was cut into a circular shape (6.42 cm^2) to serve as the e-scaffold, and a smaller circular carbon fabric "patch" (2.14 cm^2) was used as the counter electrode. The counter electrode was attached to the e-scaffold using a thin layer (~1 mm) of silicone rubber (DAP Dynaflex 230 Premium Indoor/Outdoor Sealant, catalog #18357), which provided insulation between the electrodes while still allowing oxygen to diffuse to the bottom surface of the e-scaffold for H_2O_2 generation.

For the controlled generation of H_2O_2, precise, accurate control of the potential of the e-scaffold is essential (Istanbullu et al. 2012), and this was achieved using a Gamry Series G 300™ potentiostat (Gamry Instruments, Warminster, PA, USA) in conjunction with a saturated Ag/AgCl reference electrode (Lewandowski and Beyenal 2013). Ti wires (0.025 Ti, Malin Co., Cleveland, OH, Lot #27567) were used as external connections to the potentiostat (Figure 1), and the connection resistance was consistently $<2\ \Omega$. The e-scaffold was overlaid onto biofilms grown *in vitro* on glass surface (Figure 1). This configuration allowed the ventral surface of the e-scaffold to be exposed directly to biofilms.

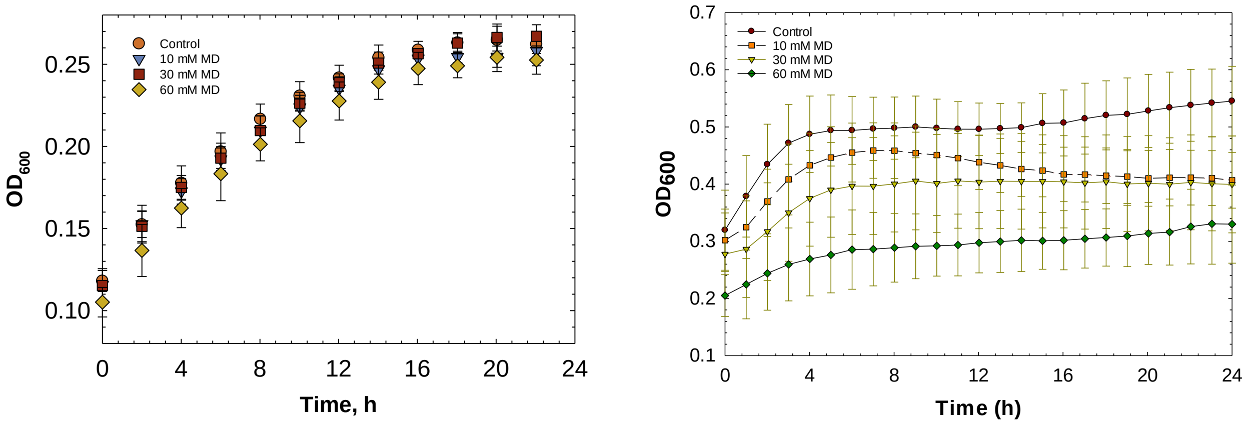

A **B**

Fig. S 1. Growth curve of A) *A. baumannii* and B) *S. aureus* in presence of maltodextrin

References

Istanbullu O, Babauta J, Hung Duc N, Beyenal H. 2012. Electrochemical biofilm control: mechanism of action. Biofouling 28(8):769-778.

Lewandowski Z, Beyenal H. 2013. Fundamentals of Biofilm Research. Boca Raton, FL: CRC Press.

Sultana ST, Atci E, Babauta JT, Mohamed Falghoush A, Snekvik KR, Call DR, Beyenal H. 2015. Electrochemical scaffold generates localized, low concentration of hydrogen peroxide that inhibits bacterial pathogens and biofilms. Scientific Reports 5:14908.
